# Integrative single-cell and bulk transcriptome analyses identify a distinct pro-tumor macrophage signature that has a major prognostic impact on glioblastomas

**DOI:** 10.1007/s10238-024-01454-5

**Published:** 2024-08-13

**Authors:** Peilin Li, Guolei Su, Yinglin Cui

**Affiliations:** 1 Second Clinical Medical College, Henan University of Traditional Chinese Medicine, Zhengzhou, 450002 China; 2 The Second Affiliated Hospital of Henan University of Traditional Chinese Medicine, Zhengzhou, 450002 China

**Keywords:** Glioblastoma, Single cell, Macrophage, S100A9, Signature

## Abstract

Glioblastoma (GBM) is a highly heterogeneous disease with poor clinical outcomes. To comprehensively dissect the molecular landscape of GBM and heterogeneous macrophage clusters in the progression of GBM, this study integrates single-cell and bulk transcriptome data to recognize a distinct pro-tumor macrophage cluster significantly associated with the prognosis of GBM and develop a GBM prognostic signature to facilitate prior subtypes. Leveraging glioma single-cell sequencing data, we identified a novel pro-tumor macrophage subgroup, marked by S100A9, which might interact with endothelial cells to facilitate tumor progression via angiogenesis. To further benefit clinical application, a prognostic signature was established with the genes associated with pro-tumor macrophages. Patients classified within the high-risk group characterized with enrichment in functions related to tumor progression, including epithelial-mesenchymal transition and hypoxia, displays elevated mutations in the TERT promoter region, reduced methylation in the MGMT promoter region, poorer prognoses, and diminished responses to temozolomide therapy, thus effectively discriminating between the prognostic outcomes of GBM patients. Our research sheds light on the intricate microenvironment of gliomas and identifies potential molecular targets for the development of novel therapeutic approaches.

**Supplementary Information:**

The online version contains supplementary material available at 10.1007/s10238-024-01454-5.

## Introduction

Glioblastoma (GBM), a prevalent malignant tumor in the central nervous system, accounts for approximately 100,000 new diagnoses worldwide annually [1]. Despite advancements in diagnostics and treatments, GBM’s high mortality and morbidity rates present ongoing challenges [2–4]. The tumor’s unique microenvironment offers insights into its progression, with macrophages and microglia key components ubiquitously interacting with GBM cells via immune mediators both within and surrounding the tumor [5, 6]. Thus, exploring their influence on GBM progression and patient prognosis is of paramount importance.

Single-cell sequencing technologies have unveiled new perspectives for dissecting the intricate cellular interactions within tumor microenvironments [6, 7]. Nonetheless, the absence of specific markers and the considerable plasticity of cells present substantial obstacles in accurately delineating their functions and molecular diversity in human cancers. Research has shown a direct correlation between the infiltration of M2-type tumor-associated macrophages (TAMs) in the GBM microenvironment and patient outcomes [5, 8]. Recent studies indicate that the M1/M2 statuses defined in vitro may not accurately reflect the true in vivo conditions [9]. Additionally, myeloid cells and the GBM cells secrete cytokines and metabolites, impeding cytotoxic T cell functions [10]. However, the detailed role and regulatory mechanisms of TAMs in GBM are still not fully understood.

This study adopts an integrative methodology, combining single-cell and bulk transcriptome analyses, to investigate the heterogeneity and functional statuses of TAMs within GBM. Our objective is to pinpoint pro-tumor macrophage subsets that are significantly associated with GBM patient prognoses. By developing a prognostic signature centered on these pro-tumor macrophages and incorporating prior molecular classifications and genomic characteristics, we comprehensively examine the signature’s biological relevance and its implications for patient outcomes. This research is poised to introduce novel strategies for both the therapeutic intervention and prognostic evaluation of GBM.

## Method

### Patient and data collection

The single-cell sequencing data for GBM were collected from the GEO database (GSE182109), with the following inclusion criteria [11]: (1) primary tumors; (2) data derived from the 10X Genomics platform; and (3) samples must contain both immune and nonimmune cell types. A total of 10 primary glioma samples were selected for analysis. Additionally, high-throughput transcriptome (RNA-seq) data, related clinical information, and multi-omics data for gliomas were downloaded from The Cancer Genome Atlas (TCGA) database. Furthermore, microarray transcriptome data for glioblastoma were collected from the GEO database, including cohorts with complete prognostic information. The inclusion criteria were: (1) primary tumors; (2) available overall survival prognostic information; (3) IDH wild-type; and (4) cohorts with more than 100 samples. Based on these criteria, three cohorts with prognostic information (GSE108474 (*n* = 219), GSE130411 (*n* = 223), GSE16011 (*n* = 95)) were selected, encompassing 537 samples, for constructing and validating the model. All cohort information is referenced in Supplementary Table S1.

### Single-cell data processing and normalization

Standard quality control measures were implemented for independent analysis: (1) Raw expression matrices from single-cell datasets were transformed into Seurat objects using the Seurat package (version 4.4.0). (2) The DoubletFinder package (version 2.0.3) was utilized to identify and remove potential doublet contamination. (3) Cells were excluded if their nFeature counts were below 200 or above 5000, or if their nCount was below 500 or exceeded 40,000, deeming them as low quality. Furthermore, cells with mitochondrial gene expression exceeding 20% were also discarded. (4) Genes expressed in fewer than five cells were eliminated. (6) For further analysis, only samples expressed in over 1000 cells were considered. After completing quality control procedures, SCTransform and RunUMAP within Seurat were employed for data normalization and dimensionality reduction, respectively. Subsequently, clustering was performed using the FindClusters function, leveraging the shared nearest neighbor optimization algorithm with a resolution parameter of 2.0. This process mapped each cell cluster into a two-dimensional space, according to dimensionality reduction outcomes, thus aiding in the annotation and identification of cell types for further analyses.

### Cell subgroup identification and characterization

Leveraging published single-cell studies, we compiled a comprehensive list of cell type-specific marker genes for annotating cell subpopulations [12–14]. GBM cells are identified by markers such as PDGFRA, SOX2, OLIG1, NES, GFAP, S100B, EGFR, MBP, and PDGFRB; endothelial cells by ACTA2, PROM1, PECAM1, TEK, and P2RY12; B cells by CD79A and IGHG1; T/NK cells by CD3D, CD3E, CD8A, CD4, and NKG7; myeloid-derived cells by CD68, S100A8, ITGAM; and oligodendrocytes by MBP and PDGFRB. Moreover, within the myeloid lineage, we differentiated between microglia, characterized by PTPRC, TMEM119, CX3CR1, and macrophages, distinguished by CD68, CD163, and MSR1.

### Functional enrichment analysis

Leveraging the MsigDB database (https://www.gsea-msigdb.org/gsea/msigdb), we utilized the clusterProfiler package [15] to perform Over-Representation Analysis (ORA) and Gene Set Enrichment Analysis (GSEA), the two leading techniques for functional enrichment analysis. The order of genes of each subtype was sorted decreasingly by logFC to serve as input files, and pathways with larger NES values were selected for visual display. To increase the persuasion of the results, we scored each sample’s pathway by another gene set enrichment algorithm, the gene set variation analysis (GSVA) [16]. These methodologies allowed us to pinpoint significantly enriched biological functions and to effectively visualize them.

### Single-cell pseudotime analysis

Single-cell pseudotime analysis techniques uncover the dynamic processes of cell differentiation within single-cell RNA sequencing (scRNA-seq) data. In our study, the R package Monocle (2.30.0) was employed to delineate a dynamic trajectory of fibroblast subgroup differentiation. Throughout this analysis, cells are allocated pseudotime values that mirror their sequential positioning along the differentiation pathway. To pinpoint potential progenitor cell populations, we utilized the R package CytoTRACE (0.3.3) to analyze single-cell gene expression data, thereby forecasting the developmental potential of individual cells.

### Cell communication

In our research, we utilized CellChat 1.1.3 to analyze normalized scRNA-seq data, which had been preprocessed using the Seurat package. The goal was to decipher and quantify the intricate web of intercellular communication among various cell types in GBM samples. Through the evaluation of key receptor-ligand pairs and their respective roles in signaling pathways, we successfully pinpointed pivotal pathways and identified the principal senders, receivers, intermediaries, and influencers operating within these networks. The analysis was conducted using the default settings of the software. We deemed findings statistically significant at *p* ≤ 0.05, applying the Benjamini–Hochberg (BH) method for correction.

### Identification of key transcription factors

In this research, pySCENIC [17] was utilized to uncover and explore critical transcription factors and their regulatory networks in macrophages. Gene expression matrices served as the initial input for the pySCENIC analysis, wherein the GRN module deduced associations between transcription factors and their potential target genes through co-expression patterns. Following this, the cisTarget reference databases were employed to pinpoint conserved gene regulatory elements adjacent to transcription factor binding sites, thus delineating direct regulatory connections between transcription factors and their targets. The AUCell module then evaluated the activity level of these transcription factor regulatory networks in individual cells, shedding light on the roles of specific transcription factors across macrophage clusters.

### Model construction and validation

**Fig. 1:**
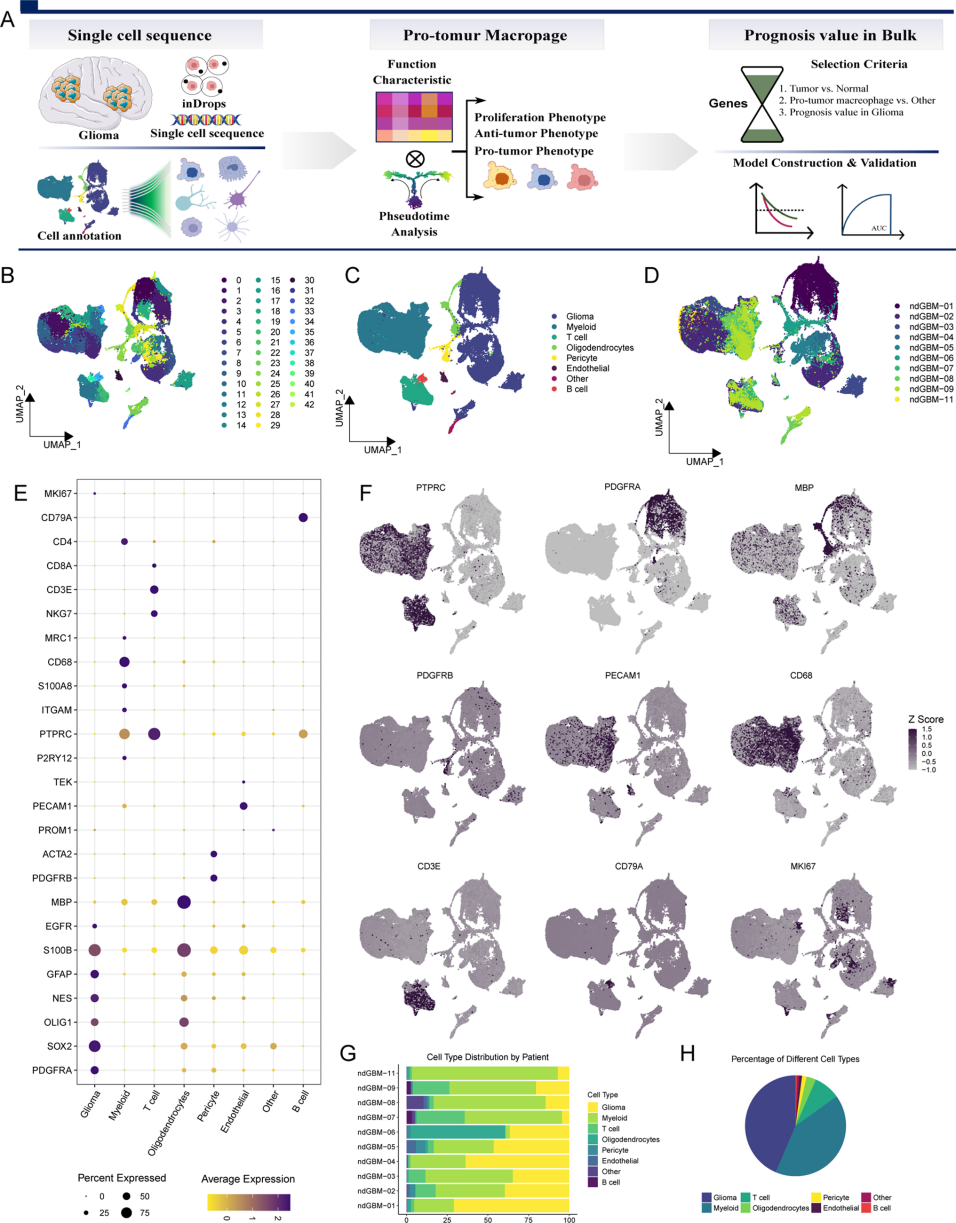
Single-cell analysis of glioblastoma (GBM). **A**. Flowchart displays analytic review of single-cell sequencing data, pinpointing of pro-tumor macrophages, and the establishment and construction of a prognostic model. **B**. Uniform manifold approximation and projection (UMAP) visualizes 93,811 cells across 43 distinct clusters. Individual cells are depicted as dots, with varying hues representing disparate cellular conglomerates. **C**. UMAP Represents detailed annotations for varied cellular clusters. **D**. Stratification of cellular distribution according to patient origins.** E**. Gene expression profiling via bubble chart. This visual delineates the expression patterns of pivotal genes across various cell typologies, where the bubble’s diameter signifies the number of cells expressing a particular gene, and its color reflects the gene’s mean expression value. **F**. UMAP showcases the differential expression of key genes among cell types. **G**. Comparative bar graph illustrating patient-wise distribution of cellular varieties. **H**. Pie Chart overview of cellular composition by type

For model construction, we selected genes based on: (1) differential expression between tumor and normal tissues; (2) relevance to prognosis; and (3) association with pro-tumorigenic macrophages. The least absolute shrinkage and selection operator (LASSO) Cox regression method was employed to streamline model complexity through a penalty term, isolating genes of utmost prognostic significance. Utilizing these genes, we developed a multivariate Cox regression prognostic model in TCGA cohort, employing a backward stepwise algorithm. This model underwent validation on three independent datasets (GSE108474, GSE13041, GSE16011), and its prognostic accuracy was evaluated by comparing survival disparities between designated high- and low-risk groups (Fig. 1A).

### Drug prediction

Sequencing and drug response data for GBM cell lines were obtained from the Cancer Cell Line Encyclopedia (CCLE, https://sites.broadinstitute.org/ccle) and the Genomics of Drug Sensitivity in Cancer (GDSC, https://www.cancerrxgene.org/) databases. Drug sensitivity was quantified using IC50 values, with lower IC50 values indicating greater sensitivity to the drug. According to a prior study [18], we then employed the ridge regression model found in the pRRophetic package, a technique previously utilized in several studies. This model, trained on expression profiles and drug response data from GBM cell lines, was used to forecast drug responses in clinical samples by leveraging GBM-specific mRNA expression profiles.

### Statistical methods

Data processing, statistical analysis, and graphical plotting were executed in R (4.3.2) and Python (3.7). The Shapiro–Wilk test was utilized to assess distribution normality, while Levene’s test was applied to evaluate variance homogeneity. Continuous variable comparisons between two groups were conducted using either the Wilcoxon rank-sum test or the *T*-test, depending on the data distribution. The relationship between two continuous variables was determined using either Spearman’s or Pearson’s correlation coefficient, based on the data characteristics. Survival analysis and Kaplan–Meier curve generation were performed using the survival and survminer packages. P-values were adjusted to false discovery rates (FDRs) employing the Benjamini–Hochberg (BH) method to account for multiple comparisons. All statistical tests were bidirectional, and a P-value of less than 0.05 was deemed indicative of statistical significance.

## Results

### GBM single-cell atlas

Following the application of rigorous quality control criteria, we isolated 93,811 cells. For PCA, we selected 3,000 genes with high variability, and then proceeded to perform UMAP analysis leveraging the top 30 principal components (PCs) in the GSE182109 dataset. This analysis resulted in the categorization of all cells into 43 distinct clusters (Fig. 1B). Utilizing marker gene expression for reference, we annotated cell types and merged analogous cell subpopulations, ultimately identifying eight unique cell types. These comprised 17 clusters of glioma cells, 3 clusters of oligodendrocytes, 14 clusters of myeloid cells, 4 clusters of T cells, and single clusters of endothelial cells, B cells, pericytes, and miscellaneous cells (Fig. 1C). Figure 1D displays the cell distribution across different patients, highlighting the ubiquity of immune cells and the unique distribution of tumor cells per patient. Marker gene expression for each cell type is presented in Fig. 1E, with Fig. 1F underscoring the elevated expression of signature genes PTPRC, PDGFRA, MBP, PDGFRB, PECAMQ, CD68, CD3E, CD79A, MKI67 in immune cells, myeloid cells, oligodendrocytes, endothelial cells, macrophages, T cells, B cells, and proliferative cells. Across various patients, glioma and myeloid cells were identified as predominant cell types (Fig. 1G, H), whereas the distribution of other cell types remained consistent across individuals, suggesting an absence of batch effects in this study.

### Tumor cell heterogeneity in glioma

**Fig. 2:**
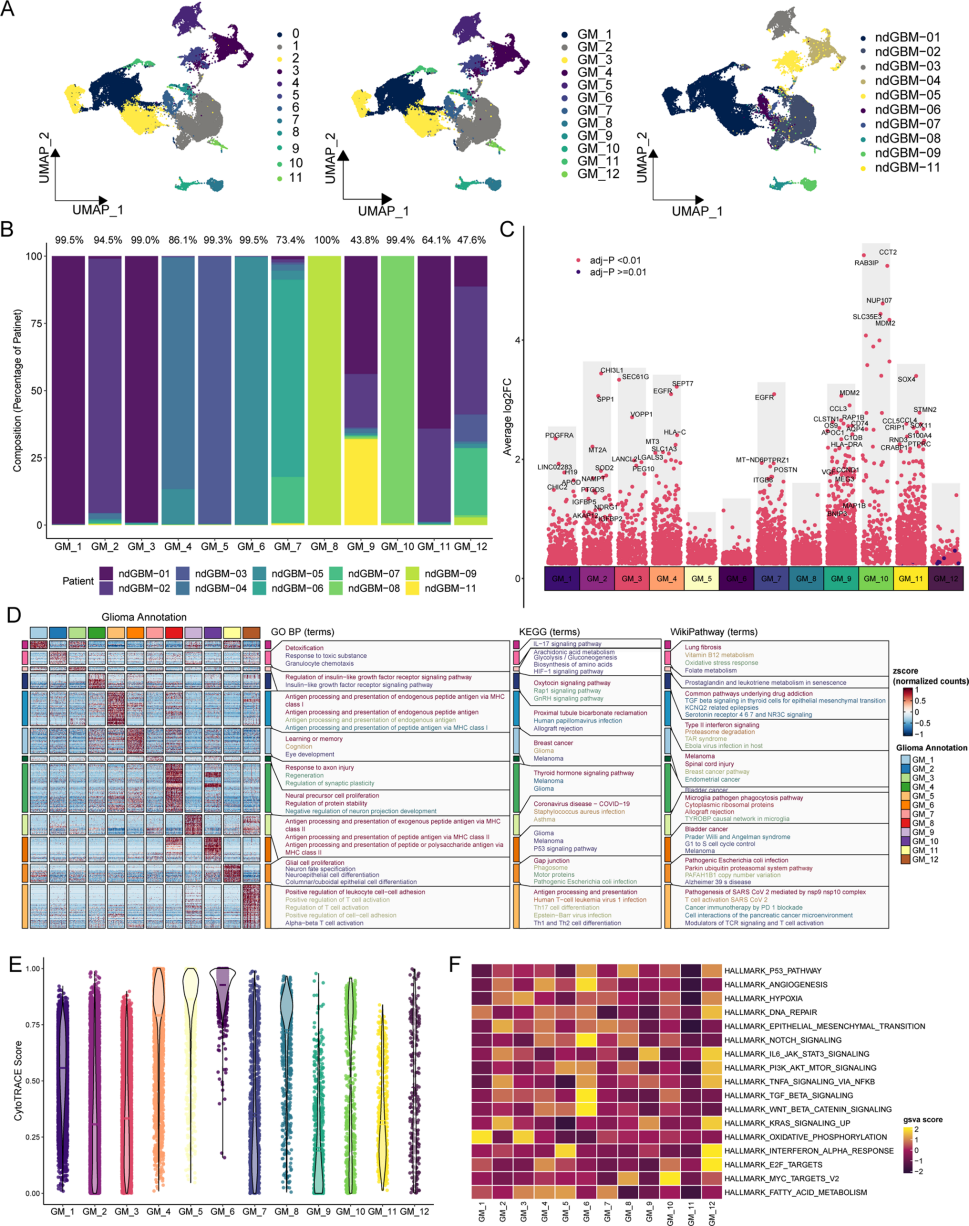
Glioblastoma Cell Subpopulations. **A**. UMAP analysis delineates GBM cells into 12 distinct subpopulations, identified as GM_1 to GM_12, with each exhibiting unique patient-derived characteristics. **B**. The bar graphs detail the proportional representation of various glioblastoma cell subpopulations across different patient samples. **C**. Manhattan plots elucidate the variances in gene expression profiles among the glioblastoma tumor cell subpopulations. **D**. Heatmaps highlight the differential gene expression across glioblastoma cell clusters and their associated functional enrichment within the GO (Gene Ontology), KEGG (Kyoto Encyclopedia of Genes and Genomes), and WikiPathways databases. **E**. Violin plots depict cytoTRACE scores to evaluate the stemness attributes of the subpopulations. **F**. Heatmaps illustrate the enrichment scores of hallmark gene sets across GBM cell clusters 1–12, providing insights into their molecular signatures

From an initial dataset of 40,864 glioma cells, reclustering was conducted to segregate these cells into 12 subgroups (Fig. 2A), labeled GM_1 to GM_12. These subgroups demonstrated patient-specific patterns, with 7 clusters representing over 90% of samples, 3 clusters representing over 60%, and 2 clusters representing over 40% (Fig. 2B). To further explore the biological traits of these glioma cell clusters, we utilized FindAllMarkers to pinpoint differential genes for each cluster, which were then visualized with Manhattan plots (Fig. 2C). This analysis of differential genes uncovered significant functional enrichments across the cell clusters, encompassing tumor proliferation, invasion, extracellular matrix remodeling, response to oxidative stress, cell cycle regulation, and immune modulation. Specifically, Fig. 2D illustrates GM_2 enriched in pathways associated with the tumor microenvironment and oxidative stress, such as the response to toxic substances; GM_3 showed predominant enrichment in pathways governing proliferation and apoptosis, notably the insulin-like growth factor receptor signaling pathway; and GM_5 to GM_12 displayed enrichment in pathways related to signal transduction activation, immune regulation, and cell cycle governance. The CytoTRACE score highlighted a notably higher stemness score for the GM_6 group (Fig. 2E). Furthermore, Hallmarker function enrichment analysis (Fig. 2F) showed consistent patterns, with GM_6 notably enriched in pathways including angiogenesis, the Notch pathway, the TGF-beta pathway, and the Wnt/beta-catenin pathway.

### *S100A9* macrophages promote tumor development through angiogenesis via interactions with endothelial cells

**Fig. 3:**
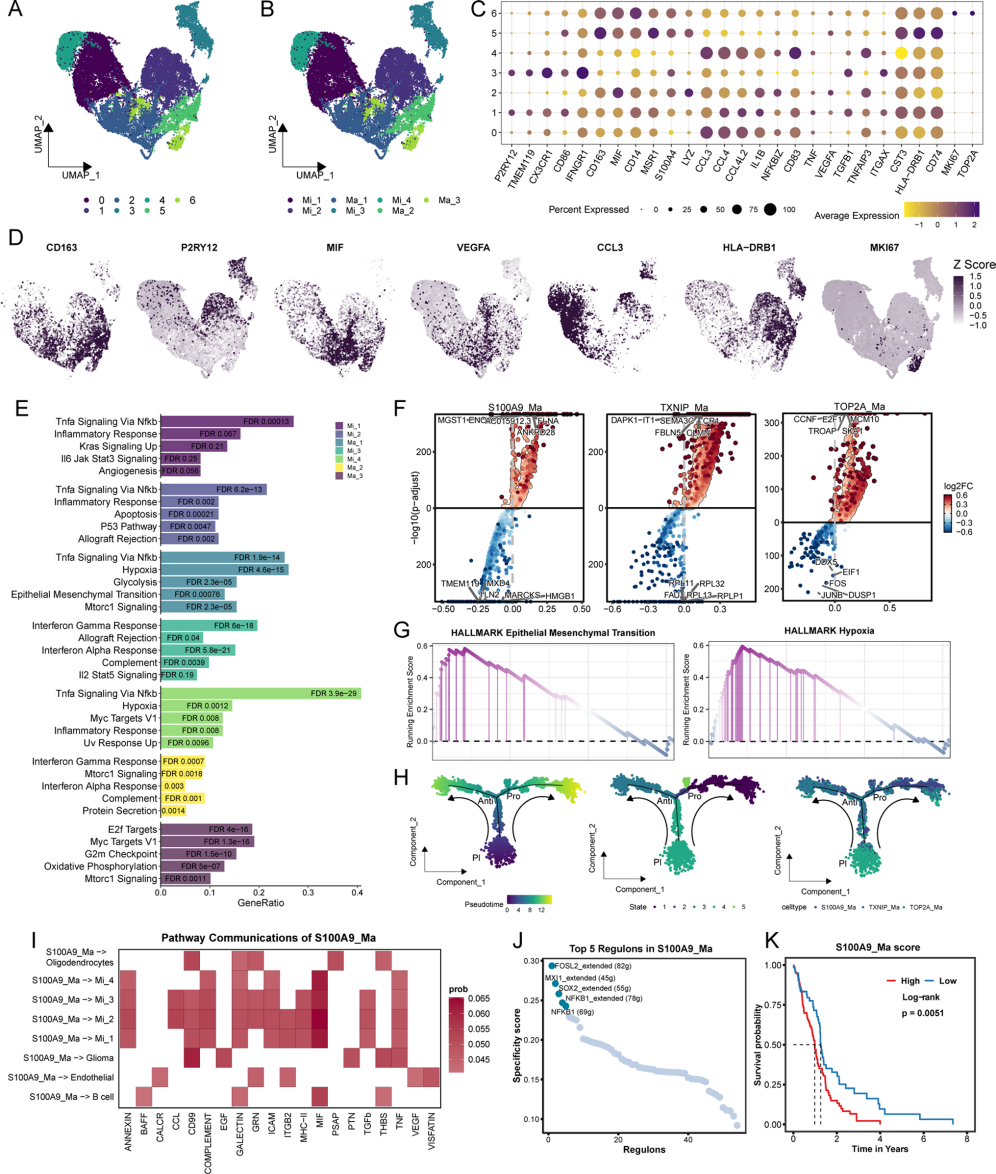
Characterization and Functional Assessment of Macrophage Clusters. **A**. UMAP delineates seven distinct myeloid cell clusters. **B**. UMAP characterizes four microglia clusters (Mi_1-4) alongside three macrophage clusters (Ma_1-3). **C**. Bubble charts detail the signature genes of macrophages, with the diameter indicating the number of cells expressing said gene and color reflecting mean expression levels. **D**. UMAP showcases the expression profiles of hallmark genes within myeloid populations. **E**. Bar graphs delineate the functional enrichment analyses for each cell cluster, employing varying colors to differentiate clusters and bar heights to signify generative processes. **F**. Volcano plots highlight the genes differentially expressed in S100A9 Ma, TXNP Ma, and TOP2A Ma, with color coding representing the logarithmic fold change (logFC). **G**. Gene Set Enrichment Analysis (GSEA) identifies pathways preferentially enriched in S100A9 Ma. **H**. Monocle trajectory plots for macrophages are sequentially colored by pseudotime, cellular state, and cell type. **I**. Heatmaps depict the interactive landscape of S100A9 macrophages with diverse cell types as analyzed through CellChat. **J**. Dot plots rank the top five transcription factors pivotal within the S100A9 macrophage subsets. **K**. Kaplan–Meier analysis underscores the prognostic disparities among patients distinguished by elevated versus diminished S100A9 macrophage infiltration levels

From an analysis of 38,784 macrophages, we performed further dimensionality reduction and clustering to delineate 7 distinct cell clusters (Fig. 3A), identifying 4 clusters as microglia and 3 as macrophages based on unique gene expression profiles (Fig. 3B) (Supplementary Table S2). The expression patterns of key marker genes such as *CD163*, *P2RY12*, *MIF*, *VEGFA*, *CCL3*, *HLA-DRB1*, and *MKI67* were meticulously charted (Figs. 3C–D). Functional enrichment analysis (Fig. 3E) and volcano plots (Fig. 3F) pinpointed the Ma_1 cluster’s enrichment in pathways pertinent to hypoxia and epithelial-mesenchymal transition, categorizing this subgroup as *S100A9* Ma, linked to tumor advancement. The Ma_2 cluster was found to be enriched in immune-related pathways, like interleukin γ and the complement system, leading to its classification as *TXNIP* Ma, associated with tumor resistance. The functional profile of Ma_3, accentuating cell proliferation pathways, defined this cluster as *TOP2A* Ma, related to cellular proliferation. GSEA corroborated our delineation of Ma_2 (Fig. 3G). Through pseudotime analysis, differentiation trajectories among subgroups were constructed, unearthing functions aligned with differentiation trends. Predominantly, *TOP2A* Ma was situated at the state and the onset of the trajectory, whereas *S100A9* Ma and *TXNIP* Ma were located at the trajectory’s conclusion (Fig. 3H). Pathway communication analysis by CellChat revealed *S100A9* Ma’s interactions with other cell types, engaging in the EGF pathway with glioma cells and the VEGF pathway with endothelial cells (Fig. 3I). In *S100A9* Ma, the upstream transcription factors *FOSL2* and *SOX2* exhibited notable regulatory activity, modulating downstream matrix metalloproteinases and cell adhesion molecules, indicators of tumorigenic activity (Fig. 3J). Further validation of *S100A9* Ma’ pro-tumor traits in bulk data, using single-sample gene set enrichment analysis (ssGSEA) in TCGA-GBM to calculate *S100A9* Ma scores, showed that patients with high infiltration scores had significantly poorer prognoses than those with low scores (Fig. 3K).

### Construction and validation of a pro-tumor macrophage-associated signature (PTM)

**Fig. 4:**
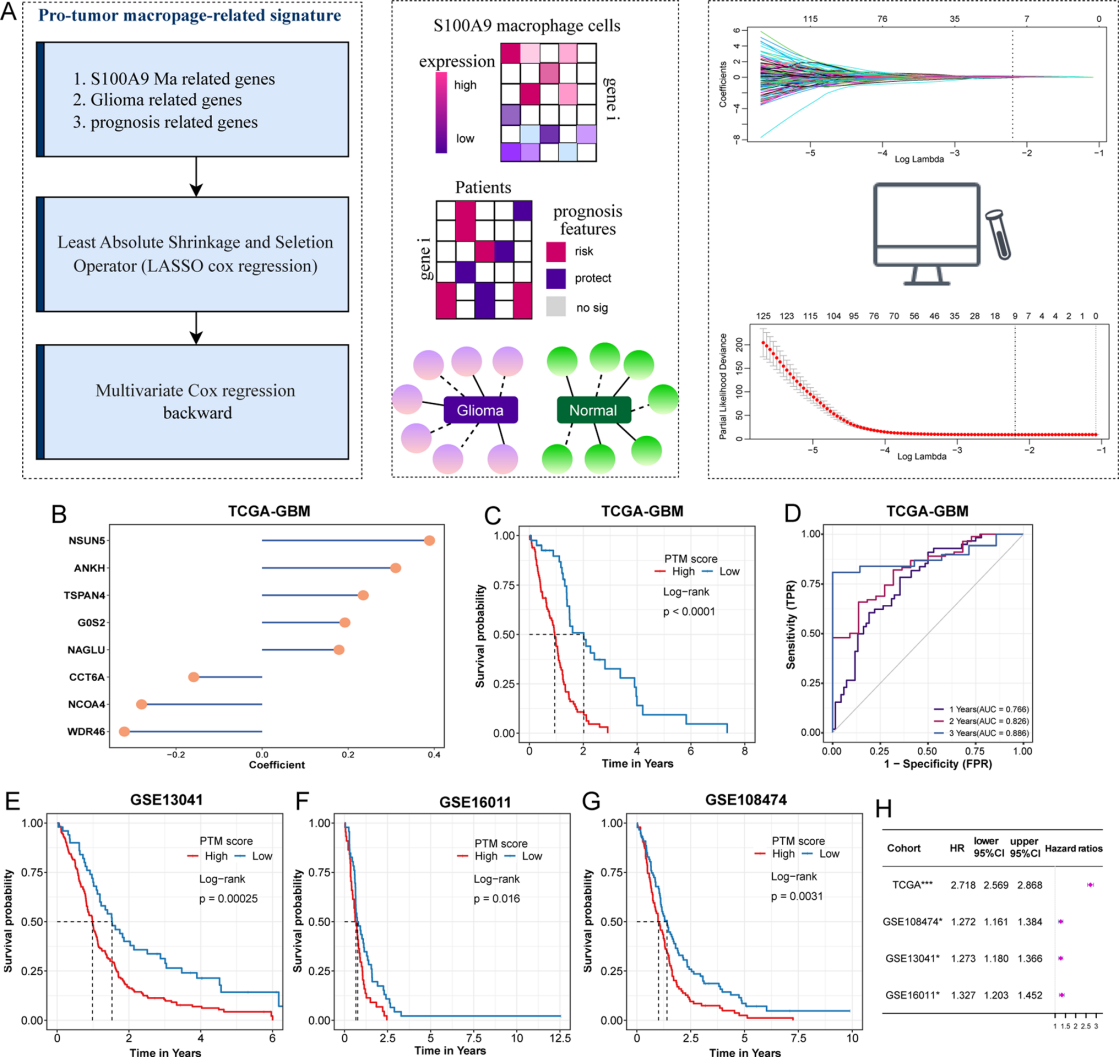
Development and Validation of a pro-tumor macrophage (PTM) prognostic model. **A**. A flowchart on the left delineates the development of a prognostic model associated with tumor-promoting macrophages in GBM patients, the center details the gene selection methodology utilized during the model’s formulation, and the right illustrates the lasso technique for gene filtration. **B**. A lollipop chart presents the eight pivotal genes incorporated into the model alongside their respective coefficients. **C**. Kaplan–Meier plots compare the outcomes of patients classified by high versus low-risk scores within the TCGA cohort. **D**. Temporal receiver operating characteristic (ROC) curves evaluate the model’s prognostic precision at 1, 3, and 5-year intervals in the TCGA training cohort. **E–G**. Kaplan–Meier plots validate the prognostic relevance of the PTM score across external cohorts: GSE108474 (**E**), GSE13041 (**F**), and GSE16011 (**G**). **H**. A forest plot synthesizes the univariate Cox regression outcomes for the PTM score, affirming its prognostic significance across both the foundational training set and subsequent validation cohorts

To construct the model, differentially expressed genes from were identified the GSE108474 cohort, which comprises both normal and tumor samples. A univariate Cox regression model was utilized to sift through genes linked with prognosis (Fig. 4A). The genes of prognostic significance were then cross-referenced with those associated with S100A9 Ma, leading to the development of a prognostic risk model through LASSO-COX regression analysis. This model ultimately featured eight genes: NSUN5, ANKH, TSPAN4, GOS2, NAGLU, CCT6A, NCOA4, and WDR46. The formula for calculating the PTM score was: NSUN5 * 0.389 + ANKH * 0.310 + TSPAN4 * 0.235 + GOS2 * 0.192 + NAGLU * 0.179 − CCT6A * 0.159 − NCOA4 * 0.280 − WDR46 * 0.320 (Fig. 4B). Leveraging the TCGA dataset as a training cohort, we stratified patients into high- and low-risk groups. Kaplan–Meier analysis showed that the high-risk group exhibited significantly reduced overall survival compared to the lower-risk group (Fig. 4C). Time ROC curve analysis for 1, 3, and 5-year survival predictions in the training set yielded the area under the curves (AUCs) of 0.766, 0.826, and 0.886, respectively. This trend was consistent in the validation cohorts, where higher-scoring patients faced poorer prognoses (GSE108474 *p* = 0.0031, GSE13041 *p* = 0.00025, GSE16011 *p* = 0.016) (Fig. 4E–G). Univariate Cox analysis affirmed that the PTM score serves as an independent risk factor, with elevated risk scores associated with increased mortality risk and reduced survival durations (Fig. 4H).

### PTM signature correlation with tumor progression

**Fig. 5:**
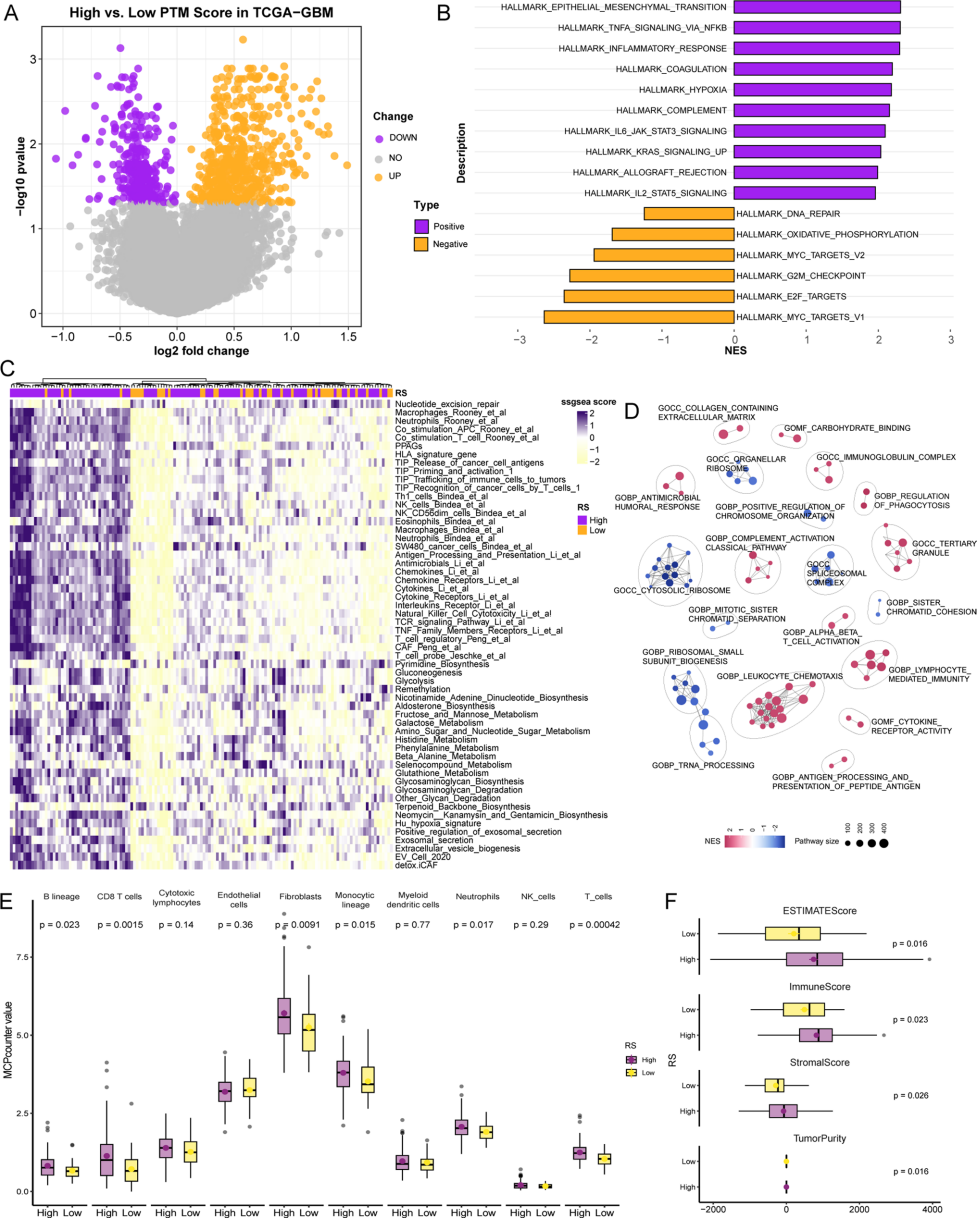
Biological significance of the PTM prognosis scoring system. **A**. A volcano plot highlights genes differentially expressed between low-risk and high-risk GBM patient cohorts within the TCGA database. **B**. A bar graph depicts hallmark gene set enrichment analysis outcomes, differentiating between high-risk and low-risk categories. **C**. A heatmap reveals the single-sample gene set enrichment analysis (ssGSEA) results, indicating functional enrichment among TCGA-GBM patient groups. **D**. A network graph illustrates the associations uncovered through functional enrichment analysis. **E**. Box plots detail the MCPcounter analysis outcomes, which quantify the extent of immune cell penetration in patient groups categorized by high and low risk. **F**. Box plots delineate the findings from the ESTIMATE algorithm, comparing immune score, stroma score, and tumor purity between high-risk and low-risk patient groups

In the TCGA dataset, differential analysis via limma was applied to separate low- and high-risk GBM patient groups (Fig. 5A). GSEA highlighted that the high-risk group was characterized by enrichment in functions related to tumor progression, including epithelial-mesenchymal transition, hypoxia, the TNFA signaling pathway, inflammatory responses, collagen synthesis, and the IL6-JAK-STAT3 signaling pathway (Fig. 5B). ssGSEA yielded similar results, with the high-score group showing enrichment in pathways linked to tumor progression, immune infiltration, and metabolic processes (Fig. 5C). By leveraging the apear package to amalgamate functions based on pathway similarities (Fig. 5D), it was found that upregulated pathways in the high-score group predominantly centered on immune regulation, antigen presentation, and the extracellular matrix, while downregulated functions concentrated on ribosomal components, organelles, and chromosomal remodeling. Consequently, we used MCPcounter to evaluate changes in immune cell infiltration across high- and low-risk groups (Fig. 5E). Box plots indicated enhanced infiltration of T cells, CD8 + T cells, and monocytes in the high-score group. Despite the observed increase in T cells, factors like T cell exhaustion and a dense matrix composition within the tumor microenvironment negatively impacted patient outcomes. The Estimate algorithm (Fig. 5F) showed that patients in the high-score group had elevated Estimate, immune, and stromal scores, providing a nuanced perspective on the prognosis for patients with elevated PTM scores.

### Clinical value of the PTM signature

**Fig. 6:**
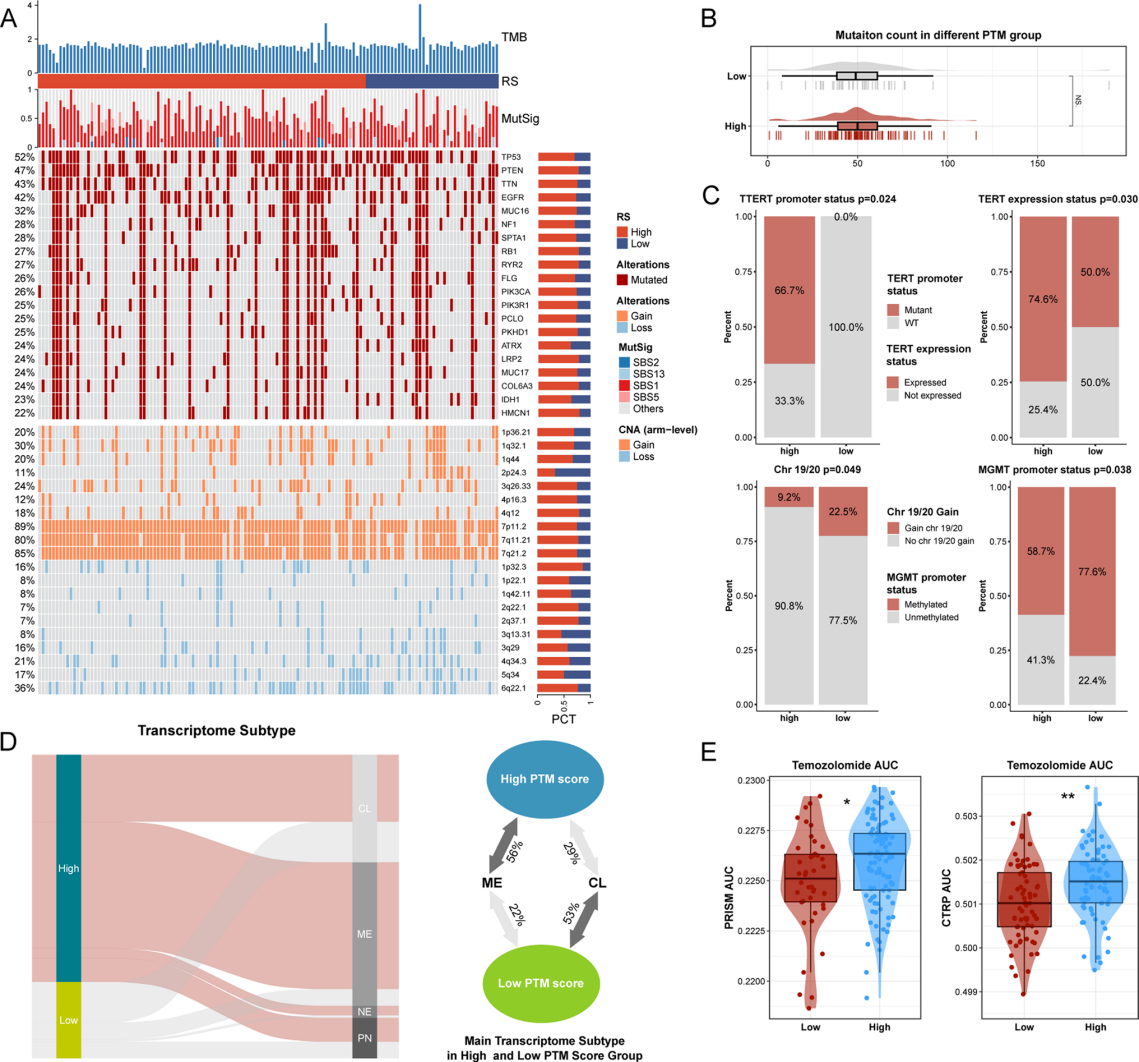
Clinical features and omics features correlated with the PTM score. **A**. Sequentially presented are the tumor mutational burden (TMB), the contributory extent of five specific mutational signatures, the leading 20 mutated genes, and copy number variations (CNAs). The distribution of high- and low-scoring cohorts relative to each genomic alteration is depicted on the right via bar charts. **B**. An evaluative comparison of mutation frequencies distinguishes between high- and low-scoring groups. **C**. Bar graphs depicting percentages delineate differences in *TERT* promoter mutations, *TERT* expression, chr19/20 amplifications, and *MGMT* promoter methylation across groups stratified by high and low PTM scores. **D**. Sankey diagram demonstrates the correlation between PTM scores and transcriptomic subtypes, accompanied by a depiction of the composition variance in transcriptomic subtypes between high and low-scoring groups on the right. **E**. Box plots reveal the area under the curve (AUC) for temozolomide efficacy within groups categorized by high versus low PTM scores. Significance indicated by *p < 0.05, **p < 0.01

GBM encompasses a comprehensive spectrum of genomic features that have been leveraged clinically to decode molecular signatures, unravel disease mechanisms, and forecast patient prognosis [19]. In this light, we delved into the potential implications of the PTM score from a genomic standpoint. Figure 6A displays the gene mutations and copy number variations (CNVs) distinguishing the high and low-score groups. Predominantly, the high-score group manifested a higher prevalence of gene mutations (including *TP53*, *PTEN*, *EGFR*, *MUC16*, *NF1*, *SPTA1*, *RB1*, *RYR2*) and CNVs, such as amplifications in 1p36.21, 1q32.1, 1q44, and deletions in 1p32.3, 2q22.1, 2q37.1, more frequently than the low-score group. These genomic changes correlate with malignant tumor traits like increased proliferation, resistance to treatment, and evasion of apoptosis [20, 21]. Although Fig. 6B revealed no significant disparity in the mean mutation count between the groups, a closer examination uncovers a consistent genomic profile in the high-score group. Importantly, a higher mutation frequency in the TERT promoter region and reduced methylation levels in the MGMT promoter area were observed in the high-score group, indicating these genomic modifications could underpin the adverse prognosis associated with higher PTM scores (Fig. 6C). Intriguingly, alterations in chromosomes 19/20 were less common in the high-score group, and these genomic features might partly explain the favorable prognosis in the low-risk score group [22]. The PTM score aligns with prior typing, with the MES subtype making up 56% of the high-score patients, while 53% of the low-score group fell into the CL category (Fig. 6D). Additionally, the PTM score was shown to have prognostic significance in determining treatment outcomes. Utilizing data from the prism and CTRP databases, the low-score group exhibited lower AUC values, signifying a heightened sensitivity to temozolomide therapy.

## Discussion

Leveraging single-cell RNA sequencing technology, this investigation delved into the cellular heterogeneity of GBM, elucidating the distribution, functions, and interactions among diverse cell types and their subsets within the malignancy. A particular focus was placed on a pro-tumor macrophage cluster marked by *S100A9*, which is implicated in promoting angiogenesis and tumor advancement through its interactions with endothelial cells. Additionally, the construction of a prognostic signature, anchored on genes linked to pro-tumor macrophages, distinguished high-score groups by elevated mutation frequencies in the *TERT* promoter region, reduced methylation of the *MGMT* promoter, adverse prognostic outcomes, and reduced efficacy of temozolomide therapy. These insights contribute a fresh perspective to the intricate microenvironment of GBM, highlighting potential molecular targets for innovating therapeutic approaches.

In GBM, our investigation delineated eight predominant cell types, inclusive of glioma cells, myeloid cells, and T cells, among others, with glioma and myeloid cells emerging as the principal cell populations. This discovery highlights the intricate interplay between tumor and immune cells within the GBM microenvironment, corroborating prior findings that myeloid cells in glioblastomas are chiefly composed of resident oligodendroglia and blood-derived macrophages [23]. Crucially, macrophages are categorized based on their functional activation into pro-tumorigenic and anti-tumorigenic phenotypes [24]. Significantly, our analysis unveils the presence of patient-specific tumor cell traits, suggesting a variation in the genetic and microenvironmental landscapes of tumors among distinct patients.

Macrophages, critical elements within the tumor microenvironment, contribute to the advancement of GBM by supporting tumor progression [25, 26]. Our research has pinpointed S100A9 macrophages [27, 28], presenting their capability to enhance angiogenesis via interactions with endothelial cells, thus propelling tumor progression [29]. Consistent with preceding research [30], our observations indicate that macrophages facilitate vascular formation through the secretion of angiogenic factors, such as VEGF [31]. Additionally, our findings shed light on the potential involvement of S100A9-positive macrophages in fostering tumor immune evasion, offering valuable perspectives on the immunoregulatory processes operative in GBM.

The prognostic signature developed from genes linked to pro-tumor macrophages has shown significant predictive capacity, indicating that the overall survival of patients classified within the high-risk category is markedly reduced compared to those in the low-risk category. Through the comparative analysis of high versus low-risk cohorts and the execution of biological process enrichment analysis, we gained insights into the underlying molecular mechanisms that contribute to differences in prognosis, including epithelial-mesenchymal transition [32], hypoxia [33], and inflammatory [7]. This is consistent with prior studies [34, 35] on the molecular features and clinical outcomes associated with GBM. The examination of genomic characteristics [36] across varying risk profiles, such as TERT promoter mutations [22] and MGMT promoter methylation levels, along with their associations with treatment outcomes (for instance, the efficacy of temozolomide), highlights the critical role of tailored therapeutic strategies in GBM treatment protocols [37].

While this study has advanced the development of prognostic signatures and the delineation of tumor molecular profiles, several domains warrant further investigation.

An enhanced understanding of the molecular underpinnings governing the interactions between pro-tumor macrophages and other cellular entities, such as GBM cells and endothelial cells, is imperative. The prognostic efficacy of the PTM score demands substantiation within broader, multicenter clinical cohorts, alongside assessments of its applicability across diverse tumor types. Initiatives to forge targeted therapies against pivotal genes identified within the prognostic model, including *NSUN5* and TSPAN4, and evaluating the efficacy of these therapeutic approaches in both animal models and clinical trials are critical.

In essence, through the establishment and validation of a prognostic signature centered on genes linked to pro-tumor macrophages, this research sheds light on new dimensions of GBM’s biological traits, prognostic forecasting, and therapeutic avenues. The formulation of the PTM score not only facilitates the identification of GBM patients at elevated risk but also lays the groundwork for future personalized treatment modalities and the identification of novel therapeutic targets. With an enriched comprehension of the GBM microenvironment, we anticipate the advent of more efficacious treatment methodologies to enhance the prognosis for individuals afflicted with GBM.

## Supplementary Information

Below is the link to the electronic supplementary material.

Supplementary file1 (XLSX 180 KB)
